# *CDH1 *promoter hypermethylation and E-cadherin protein expression in infiltrating breast cancer

**DOI:** 10.1186/1471-2407-6-48

**Published:** 2006-03-02

**Authors:** José Roberto F Caldeira, Érika C Prando, Francisco C Quevedo, Francisco A Moraes Neto, Cláudia A Rainho, Silvia R Rogatto

**Affiliations:** 1 Department of Senology, Amaral Carvalho Hospital, Jaú, Sao Paulo, Brazil; 2 Department of Genetics, Institute of Bioscience, Sao Paulo State University, Botucatu, Sao Paulo, Brazil; 3 Department of Pathology, Amaral Carvalho Hospital, Jaú, Sao Paulo, Brazil; 4 NeoGene Laboratory, Department of Urology, Faculty of Medicine, Sao Paulo State University, Botucatu, Sao Paulo, CEP 18618-000, Brazil

## Abstract

**Background:**

The E-cadherin gene (*CDH1*) maps, at chromosome 16q22.1, a region often associated with loss of heterozygosity (LOH) in human breast cancer. LOH at this site is thought to lead to loss of function of this tumor suppressor gene and was correlated with decreased disease-free survival, poor prognosis, and metastasis. Differential CpG island methylation in the promoter region of the *CDH1 *gene might be an alternative way for the loss of expression and function of E-cadherin, leading to loss of tissue integrity, an essential step in tumor progression.

**Methods:**

The aim of our study was to assess, by Methylation-Specific Polymerase Chain Reaction (MSP), the methylation pattern of the *CDH1 *gene and its possible correlation with the expression of E-cadherin and other standard immunohistochemical parameters (Her-2, ER, PgR, p53, and K-67) in a series of 79 primary breast cancers (71 infiltrating ductal, 5 infiltrating lobular, 1 metaplastic, 1 apocrine, and 1 papillary carcinoma).

**Results:**

*CDH1 *hypermethylation was observed in 72% of the cases including 52/71 ductal, 4/5 lobular carcinomas and 1 apocrine carcinoma. Reduced levels of E-cadherin protein were observed in 85% of our samples. Although not statistically significant, the levels of E-cadherin expression tended to diminish with the *CDH1 *promoter region methylation. In the group of 71 ductal cancinomas, most of the cases of showing *CDH1 *hypermethylation also presented reduced levels of expression of ER and PgR proteins, and a possible association was observed between *CDH1 *methylation and ER expression (p = 0.0301, Fisher's exact test). However, this finding was not considered significant after Bonferroni correction of p-value.

**Conclusion:**

Our preliminary findings suggested that abnormal *CDH1 *methylation occurs in high frequencies in infiltrating breast cancers associated with a decrease in E-cadherin expression in a subgroup of cases characterized by loss of expression of other important genes to the mammary carcinogenesis process, probably due to the disruption of the mechanism of maintenance of DNA methylation in tumoral cells.

## Background

Breast cancer is the second leading cause of cancer-related morbidity and mortality in women worldwide. In 2005, more than 49,500 new cases of breast cancer among Brazilian women are expected. Despite the recent trend in decreasing in mortality rates, probably due to improvements in early detection, approximately 50% of cases were diagnosed as advanced disease (grade III and IV tumors) in recent years in our country [1].

The natural history of breast cancer is characterized by heterogeneity within and between patients, since tumors with similar histopathological diagnosis can follow different clinical courses and show different responses to therapy [2]. These cancers are generally considered to result from the accumulation of multiple clonal changes in genes that regulate cell growth and differentiation [3,4].

One of the mechanisms involved in the carcinogenesis process is loss of tumor suppressor gene function, which normally acts as a negative regulator of cell proliferation. Tumor suppressor gene inactivation contributes to carcinogenesis by conferring certain advantages to growth that lead to tumor progression. It is generally thought that these genes are recessive, requiring mutation or loss of both alleles for functional inactivation. The loss of heterozygosity (LOH) in sporadic cancers reflects somatic deletions that involve specific chromosomal regions associated with a tumor suppressor gene located within the deleted region [5].

Among the genome regions that undergo LOH in breast cancer is the long arm of chromosome 16 [6-8]. Using radiation hybrid mapping and microsatellite markers, Chalners et al [9] showed a high rate of LOH involving the cluster of cadherin genes at 16q21-q22.1, with the *CDH1 *gene being the most frequent lost marker in breast cancer.

The *CDH1 *(16q22.1) gene encodes the transmembrane glycoprotein E-cadherin that is important in maintaining homophilic cell-cell adhesion in epithelial tissues [10]. The cadherins are a family of Ca^2+ ^dependent adhesion molecules that function in cell recognition and tissue morphogenesis. Alterations in E-cadherin expression have been related in several cancer types and correlated with pathological features such as poor tumoral differentiation, infiltrative growth, lymph node metastasis and decreased patient survival [11-13].

Compelling experimental evidence indicates that the *CDH1 *gene is a tumor suppressor. Germ line mutations in this gene predispose affected individuals to diffuse-type gastric cancer and it was also detected in one family with a history of diffuse gastric cancer and early-onset breast cancer [14]. Further evidence has been offered by the identification of somatic inactivating mutations. In sporadic infiltrating lobular carcinomas (ILCs), most mutations found in the *CDH1 *gene were out-of-frame, which were predicted to yield secreted truncated E-cadherin fragments. In most cases these mutations occurred in combination with loss of the wild type allele [15-17]. The complete absence of E-cadherin expression, due to the presence of classical inactivating mutations and deletions, is a feature of ILCs [18].

While immunohistochemical studies have demonstrated that reduced or absent E-cadherin expression is also common in infiltrating ductal carcinomas (IDCs) [18,19], in the majority of these cases, *CDH1 *mutations were rare or absent. Recently, it was demonstrated that epigenetic silencing of the gene *CDH1 *by CpG island methylation of its promoter region, occurs in some human breast cancer cell lines, as well as in unselected primary breast cancer [20,21]. Nass et al [22] demonstrated that hypermethylation of the *CDH1 *promoter region was evident in 30% of *in situ *ductal carcinomas and increased to 60% in IDCs. In this study, we reported aberrant methylation of the 5'CpG island of the *CDH1 *gene associated with reduced levels of E-cadherin expression in breast cancer.

## Methods

### Patients

Seventy nine breast carcinoma samples (71 IDCs, 5 ILCs, 1 apocrine, 1 metaplastic, and 1 papillary) were obtained from seventy nine patients at Amaral Carvalho Hospital, Jaú, São Paulo, Brazil from 2000 to 2004. The patients were accrued consecutively and the inclusion criteria were no previous new histologic diagnosis of breast cancer. The patients had undergone segmental resection or mastectomy and none of the patients had received radiotherapy or chemotherapy before surgery. All the patients were advised of the procedures and provided written informed consent. This study was approved by the Brazilian Ethics Committee (CONEP 694/1999). Patients were regularly followed postoperatively. Immediately after surgery, the tumor samples were frozen at -80°C. Each sample was histopathologically evaluated to ensure the presence of at least 80% of tumoral cells. The medical records of all patients were examined to obtain clinical and histopathological information. The family history of cancer considered first-degree and second-degree relatives with cancer as informative, and whenever possible, the evidence of cancer was based on documented medical records or ascertained from the death certificate. The histopathological diagnoses of the tumors were described according to the WHO International Classification of Disease for Oncology [23]. The clinical staging was determined by the TNM Staging System [24]. The malignancy of infiltrating carcinomas was scored according to the Scarff-Bloom-Richardson classification [25].

### Immunohistochemical analysis

Standard immunohistochemical detection, with minor modifications, was performed on sections from archival paraffin embedded tissue. Protein expression was studied using specific antibodies against the estrogen receptor (ER) (clone RTU-ER-6F11, Novocastra, Newcastle, UK, 1:50 dilution), the progesterone receptor (PgR) (monoclonal mouse anti-human progesterone receptor 1^A^6, Dako, Carpinteria, CA, USA, 1:50 dilution), p53 (monoclonal mouse anti-human p53 protein clone DO-7, Dako, dilution 1:200), Ki-67 (monoclonal mouse anti-human ki-67 antigen clone MIB-1, Dako, dilution 1:100), Her-2 (policlonal rabbit anti-human cerbB-2 oncoprotein, Dako, dilution 1:800) and E-cadherin (monoclonal mouse anti human E-cadherin NCH-38^4^, Dako, dilution 1:25). Positive and negative controls for each marker were routinely performed during experiments. Slides were distributed randomly to two independent blinded observers (FCQ and FAMN). If any discrepancies between classifications of samples arose, they were reviewed and the final results reached by consensus. In areas of well-preserved tissue, the fraction of the infiltrating part of the neoplasm was scored. Tumors were classified by intensity of staining and the percentage of cells showing antibody reactivity. The ER, PgR, and p53 sections were scored for the immunohistochemical signal as follows: weak (1+), moderate (2+), and strong (3+) staining in >10% of the tumor cells or absent (0). The Ki-67 sections were scored for the percentage of cells stained. For E-cadherin and Her-2, the strength of the membranous staining was recorded by a four-step scale 0, 1+ to 3+ as follows: continuous staining of membrane (3+), continuous staining present in >10% of the tumor cells (2+), focal or discontinuous staining present in >10% of tumor cells (1+), and staining in <10% of infiltrating cells (0). Further, for E-cadherin expression we evaluated the staining in the cell membrane and in the cytoplasm.

### DNA extraction

Genomic DNA was prepared from frozen tumor tissues by standard SDS/proteinase K digestion followed by phenol and chloroform extraction and ethanol precipitation.

### Treatment with sodium bisulfite

The conversion of DNA by sodium bisulfite was performed using an established protocol [26], with modifications. Initially, 1–2 μg of genomic DNA were denatured with 2 M NaOH at 50°C for 20 min (final concentration of 0.2 M NaOH), followed by incubation with freshly prepared 2.5 M sodium bisulfite/1 M hydroquinone, pH 5.0, in a total volume of 520 μl, at 70°C for 3 h. The DNA was purified with the Wizard DNA Clean-UP System (Promega. Madison, WI, USA). The modification of the DNA was completed by the addition of 5.0 μl of NaOH 3 M at room temperature during 10 min. The precipitation was carried out through the addition of 75 μl of ammonium acetate 5 M (pH 7,0), 350 μl of ethanol and 1.0 μl of glycogen (20 μg/μl) (Invitrogen, Life Technologies, Carlsbad, CA, USA). The bisulfite-modified DNA was resuspended in 30 μl of sterile water, and stored at -20°C.

### Methylation-Specific Polymerase Chain Reaction (MSP)

The methylation pattern within the CpG island in exon 1 of the *CDH1 *gene (sequence -126 bp to +144 bp relative to transcription start, GenBank accession number D49685) was determined using a nested-PCR approach that has been published previously [27]. In the first round of PCR, bisulfite-treated DNA was amplified using the primers 5'-GTTTAGTTTTGGGGAGGGGTT-3, (sense) and 5'-ACTACTACTCCAAAAACCCATAACTAA-3' (antisense). The cycling conditions consisted of an initial denaturation step at 95°C for 5 min, followed by 35 cycles of 95°C for 1 min, 50°C for 30 sec, 72°C for 1 min. The size of the product after this initial PCR reaction was 270 bp. For the second PCR, 5 μl of this product was used for MSP. The nested primers for the methylated sequence reaction were 5'-TGTAGTTACGTATTTATTTTTAGTGGCGTC-3' (sense) and 5'-CGAATACGTCGAATCGAACCG-3' (antisense), and primers sequences for the unmethylated sequence were 5'-TGGTTGTAGTTATGTATTTATTTTTAGTGGTGTT-3' (sense) and 5'-ACACCAATACAACAAATCAAACCAAA-3' (antisense). The reactions were performed in a total volume of 25 μl containing 0.25 μM of each primer pair, 200 μM of each dNTP, 20 mM Tris-HCl, pH 8.4, 50 mM KCl, 1.5 mM MgCl_2_, and 1 U of Taq polymerase. The PCR parameters were the same listed above, except that the annealing temperature used for both primer pairs was 53°C. The product sizes of the methylated and unmethylated amplicons were 112 bp and 120 bp, respectively. The amplified products were visualized after electrophoresis in 6% polyacrylamide gel and silver nitrate staining [28]. Water blanks were included in each assay.

DNA from lymphocytes of healthy volunteers treated with *SssI *methyltransferase (New England Biolabs, Beverly, MA, USA) and then subjected to bisulfite modification was used as positive controls for methylated alleles. The reaction was performed in a total volume of 50 μl containing 10 μg of genomic DNA, 10 U of *SssI *methylase, 160 mM of S-adenosyl-metionina, 50 mM of NaCl, 10 mM of Tris-HCl, 10 mM of MgCl_2_, 1 mM of DTT pH 7.9, during 18 hours at 37°C.

### Statistical analysis

Descriptive statistics, such as mean, SD, and percentage were used to summarize a patient's data and gene hypermethylation status. Due to the large number of comparisons in the same sample group, pairwise associations between *CDH1 *methylation patterns and immunohistological markers, demographic and clinical variables were assessed via Fisher's exact test with a 5% level of significance. These pairwise tests utilized the following dichotomous variables, which were defined prior to any analysis on the basis of the staining index defined as absent/reduced expression levels (scores 0, 1, and 2) and positive/maximum staining (score 3) for E-cadherin, Her-2, ER, PgR, proteins. To p53 protein, the expression level was categorized as negative (scores 0 and 1) or positive (scores 2 and 3). The statistical tests were performed using the statistical software package SAS/STAT, version 6.0 (SAS Institute, Inc., Cary, NC, USA). The Bonferroni correction for the multiple comparisons was applied to adjust the p-value.

## Results

**Figure 1:**
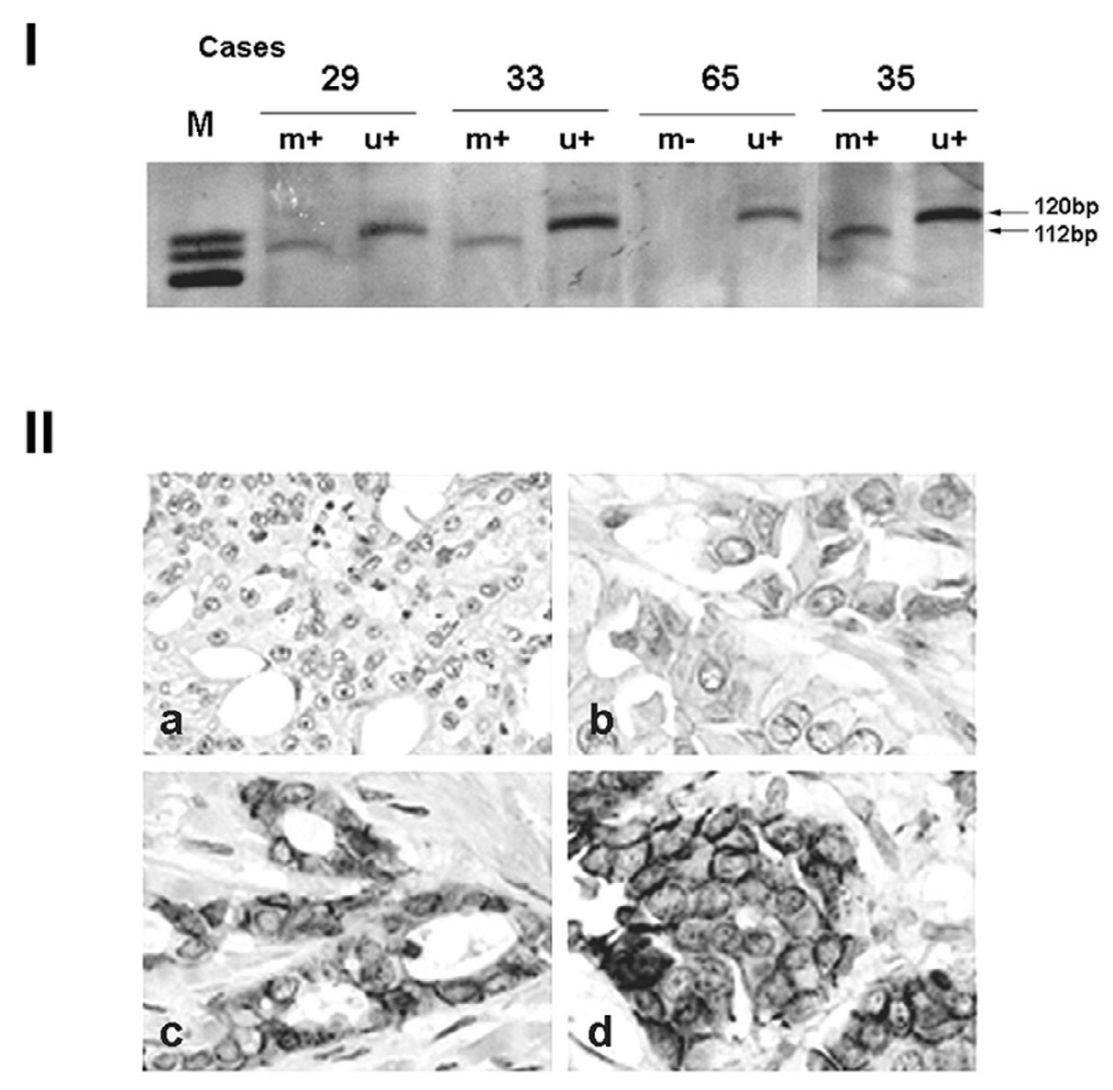
I) Representative examples of MSP for *CDH1 *gene in infiltrating ductal carcinomas. Lanes (m) and (u) correspond to reactions specific for methylated and unmethylated DNA, respectively. M, molecular weight DNA marker. II) Patterns of E-cadherin expression in ductal breast carcinomas stratified according a four-step scale: a. negative; b. 1+; c. 2+; and d. 3+. The sections were counterstained with hematoxylin. 400× magnification.

The mean age at diagnosis of the primary breast cancer was 59.6 ± 19.8 years, ranging from 30 to 94 years. The nested PCR approach was used to detect the *CDH1 *gene methylation pattern, after the treatment of tumoral genomic DNA with sodium bisulfite. In all samples, we detected the amplicon of 120 bp from unmethylated alleles. The amplification product of 112 bp from methylated alleles was observed in 52 out of 71 IDCs (73.2%), in four out of five ILCs, and in the apocrine carcinoma. The hypermethylation of the promoter of the gene *CDH1 *was detected in 11 out of 17 cases that reported positive familial history of cancer. Representative examples of MSP of the *CDH1 *gene are illustrated in Figure 1.

Due to the large number of comparisons in the same sample group, we applied the Bonferroni correction. This type of adjustment recommended a significance level of 0.005 to each of the ten tests performed. Table 1 shows the details of pairwise associations between *CDH1 *gene methylation and clinical and histological parameters in the group of 71 IDCs. No statistically significant differences in the frequencies of *CDH1 *gene promoter methylation were found between the patients at ages ≤ 50 and > 50 years old; lymph nodes status (more or less than 4 positive lymph nodes); clinical stages, histological grade and immunohistochemical analysis.

**Figure 2:**
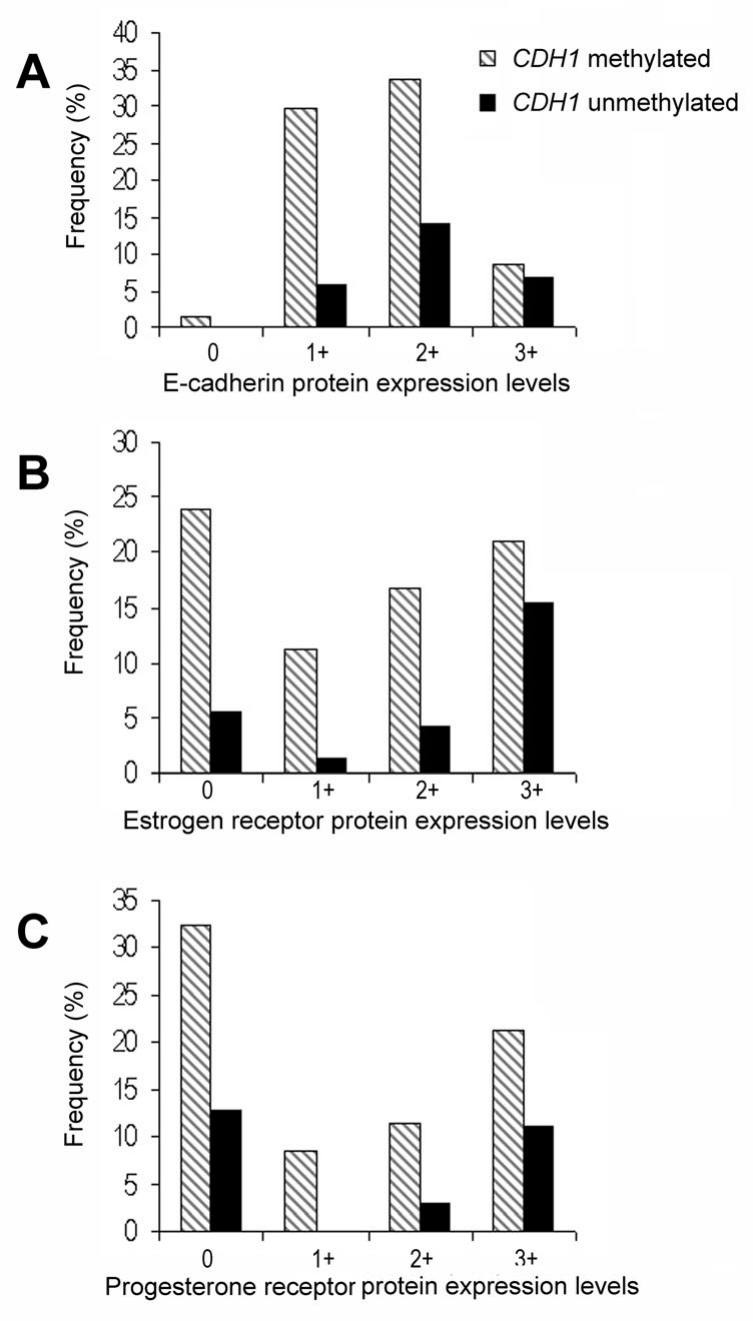
Frequency distribution of the *CDH1 *methylation pattern in 71 infiltrating ductal carcinomas, categorized by the protein expression levels of A) E-cadherin, B) ER and, C) PgR, respectively.

In the group of IDCs, *CDH1 *promoter methylation was found in 1 tumor showing absence of E-cadherin expression and in 45 weakly positive tumors (scores 1+ and 2+). Discrepant data was observed in six IDCs that showed intense staining for E-cadherin expression (score 3+) and concomitant *CDH1 *methylation. All these six cases also presented inflammatory cells, with intense (2 cases), moderate (2 cases), and discrete (2 cases) leukocytes infiltration. Of the unmethylated cases, five were E-cadherin positive and 14 showed reduced levels of expression (Figures 1 and 2).

We also compared the methylation pattern of the *CDH1 *gene and its possible correlation with the expression of E-cadherin and other standard immunohistochemical parameters (Her-2, ER, PgR, p53, and Ki-67) in this subgroup of patients with 71 IDCs. Most of the cases with reduced ER expression (scores 0 to 2+) presented hypermethylation (37 out of 45 cases); furthermore, 11 out of 26 cases with ER expression 3+ showed unmethylated *CDH1 *(p = 0.0301, Fisher's exact test). Similarly, a high frequency of cases showing reduced levels of PgR (scores 0 to 2+) protein also presented *CDH1 *hypermethylation (37 out of 48 cases). Of the cases showing strong PgR staining, 15 were methylated and 8 were unmethylated.

On average, the patients were accompanied by a follow-up of 32 months (ranging from 12 to 59 months). During these intervals three patients died from unrelated causes. Of the methylated cases, two IDCs evolved to bone and lung metastasis after a post-surgery period of 38 and 5 months, respectively. Two other cases presented local recidive after 4 and 22 months. In the group of 19 cases negative for *CDH1 *hypermethylation, we detected two cases that presented bone and lung metastasis.

The immunohistochemical analysis showed intense E-cadherin staining in the metaplastic carcinoma, weak staining in both the papillary and apocrine carcinoma. All ILCs were negative for the expression of E-cadherin.

## Discussion

Reduced expression of E-cadherin is regarded as one of the main molecular events involved in the dysfunction of the cell-cell adhesion system, triggering cancer invasion and metastasis [29]. In our study, we correlated the hypermethylation at the *CDH1 *locus and the E-cadherin expression levels determined by immunohistological analysis in 79 unselected primary breast tumors. Based on a nested PCR approach, we detected *CDH1 *hypermethylation in 52/71 IDCs, 4/5 ILCs and in the apocrine carcinoma. Nested MSP is a two-step PCR that has the sensitivity to detect methylated DNA molecules when they comprise as little as 5% of the total complex DNA sample [30] or 1 methylated allele in the presence of 1000–2000 unmethylated alleles [31].

Aberrant methylation of the promoter region is considered one of the major mechanisms for the silencing of cancer-related genes, resulting in down-regulation of gene expression. It has been demonstrated that CpG island hypermethylation is implicated in the loss of expression of a variety of critical tumor suppressor and growth regulatory genes distributed in several categories including cell cycle regulating, steroid receptors, tumor susceptibility, carcinogen detoxification, cell adhesion and inhibitors of matrix metalloproteinases [20,21]. In breast cancer, *CDH1 *promoter methylation has been reported in ~30% of *in situ *ductal carcinomas and increased substantially to nearly 60% in metastatic tumors [22]. Furthermore, this gene is one of the most frequently inactivated by methylation in sporadic breast cancer [32], as well in DNA samples obtained from plasma of invasive breast cancer patients [33], from fine needle washings from breast lesions [34], and in sentinel lymph node metastasis [35]. These findings suggested that *CDH1 *promoter methylation is an important event associated with the pathogenesis of breast cancer.

In the present study, the methylation pattern of the *CDH1 *gene was not correlated with the age of patients at diagnosis suggesting that they are not due to age-related methylation changes [36] but probably is correlated with the deregulation of the methyltransferase activity during tumor progression [37]. Disruption of the maintenance mechanism of methylation could lead to a genomic wide effect with several CpG islands showing abnormal methylation patterns; however, this event is considered nonrandom in tumor cells, as suggested by abnormal tumor-specific methylation profiles [32]. Nass et al. [22] reported previously that coincident methylation in both CpG islands of *CDH1 *and the estrogen receptor gene increases with advancing disease, suggesting that malignant progression of ductal carcinomas involves the accumulation of multiple epigenetic hits. This association was confirmed by Perrela et al. [32] which identified other methylated genes associated with estrogen receptor promoter hypermethylation.

We observed a high frequency of cases with *CDH1 *promoter hypermethylation and reduced expression of the estrogen receptor: 45 IDCs showed a decrease in ER levels (scores 0 – 2+), and 37 of them presented *CDH1 *hypermethylation. The progesterone receptor is another gene that is also inactivated by hypermethylation [38]. *CDH1 *hypermethylation was observed in 37 out of 48 cases that showed reduced expression of PgR protein (77%). In contrast, in the group showing strong PgR staining, 15 out of 23 cases presented *CDH1 *hypermethylation. Additionally, we detected the complete absence of ER and/or PgR expression in 19 out of 52 (37%) IDCs samples showing methylation of the *CDH1 *gene. These data support the hypothesis that disruption of the maintenance mechanisms of the methylation pattern could result in distinct hypermethylation profiles of primary breast cancer, with tumor subsets characterized by reduced expression of specific cancer-related genes. In IDCs, expression studies have routinely revealed that loss of expression of E-cadherin and ER exhibits heterogeneity. Similar to our findings, early studies have demonstrated that the heterogeneous patterns of CpG island methylation parallel the heterogeneous loss of both ER and E-cadherin expression in these tumors [39,40]. Thus, it seems likely that the accumulation of epigenetic changes may contribute to the diminished expression of key genes for the mammary carcinogenesis process.

Seventeen patients reported positive familial history of cancer; six of them presented breast cancer history between first and/or second-degree relatives. The hypermethylation of the promoter of the gene *CDH1 *was detected in 11 out of these 17 cases, including all six cases with familial breast cancer history. Reduced levels of E-cadherin protein were observed in four out of six cases. Most of these cases showed decreased levels of ER and PgR protein: two cases were negative for both and reduced expression of at least one protein was detected in four tumors. Although distinct differences were observed in hypermethylation profiles in cancers occurring in the familial setting, Esteller et al. [41] reported that in *BRCA1 *tumors *CDH1 *methylation frequencies resembled sporadic breast cancer.

Several reports have demonstrated the association either of the *CDH1 *gene methylation [22,32-35] or the abnormal expression of E-cadherin in breast cancer progression [40]. However, there are a limited number of studies directly correlating *CDH1 *methylation and E-cadherin expression in the same sample of breast carcinomas [42]. In our study, *CDH1 *promoter methylation was not uniformly associated with the loss of E-cadherin expression. Overall, reduced expression of E-cadherin (moderate, weak or absence of staining in cell membranes) was observed in 85% of our samples. Although not statistically significant, the intensity of E-cadherin staining tended to diminish with methylation of the *CDH1 *promoter region: 65% of the IDCs (46 out of 71 cases) showed concomitant reduced levels of E-cadherin and *CDH1 *hypermethylation. In 14 IDCs (20%), we detected only unmethylated *CDH1 *alleles despite reduced staining for E-cadherin. Several studies have demonstrated that E-cadherin expression may be repressed by mechanisms other than promoter hypermethylation, such as allele loss (LOH), gene mutation, changes in chromatin structure [43], and alterations of specific transcription pathways regulating the expression of the *CDH1 *gene [44,45]. Besides, of the methylated tumors, 6 cases showed strong E-cadherin staining (score 3+). CpG island hypermethylation constitutes a positively detectable signal with a very high sensitivity [46], thus intratumoral heterogeneity and the sensitivity of the nested MSP approach could explain these discrepant results. Alternatively, it has been demonstrated that infiltrating leukocytes can originate a methylation specific PCR fragment, making the detection and interpretation of tumor-associated altered methylation patterns more difficult [47]. Our results support this hypothesis because intense (2 cases), moderate (2 cases), and discrete (2 cases) leukocytes infiltration was detected in these six cases.

In our study, we did not find an association between *CDH1 *methylation and the Her-2 and p53 status. Her-2 over-expression was detected in 8 IDCs, of which 5 showed *CDH1 *hypermethylation. Although an inverse correlation has been related between the E-cadherin levels and Her-2 over-expression [48], some studies have detected that E-cadherin expression persisted into later stages of the disease, and was associated with Her-2 expression [49,50]. One explanation for this discrepancy is that gene expression is diverse and the studies are conducted on a large range of patients with ethnic and socio-economic variables. In our country, most breast cancer patients are diagnosed in advanced stages. Thirty-seven out of 79 cases showed clinical and or histological parameters of worse prognostic, including histologic grade II – III, positive lymph nodes, tumor size, and high Ki-67 (> 25%). However, all patients presented a favorable outcome after more than 24 months of follow-up (25–59 months), none of them recidivated or developed metastasis. In this particular subset of high risk of breast cancer, 31 tumors showed low E-cadherin levels and *CDH1 *hypermethylation was detected in 21 of them (68%). Histopathological analysis has revealed inconsistencies in the correlation of E-cadherin expression and prognosis in breast cancer. Our data are in agreement with Howard et al. [45], who find no correlations between E-cadherin expression and overall survival in breast cancer patients.

Different mechanisms are involved in the altered E-cadherin expression seen in different subtypes of breast carcinomas. In ILCs, the complete loss of E-cadherin expression has been consistent with biallelic inactivation of *CDH1 *by promoter methylation, mutation or allelic loss in any combination [40,51]. In our study, we detected absence of E-cadherin expression in all five cases of ILCs and in four of them there was concomitant *CDH1 *promoter hypermethylation, suggesting that epigenetic changes of *CDH1 *gene occurs in breast cancer, irrespective of the histological type.

## Conclusion

In conclusion, abnormal *CDH1 *methylation occurs in high frequencies in infiltrating breast cancers. Although considered not statistically significant, in a subgroup of cases, this finding was associated with the low expression levels of others genes (*ER *and *PGR*) probably due to the disruption of the maintenance mechanism of DNA methylation in tumoral cells. Our preliminary findings needed to be confirmed by new studies. The correlation between *CDH1 *hypermethylation and the reduced expression of the estrogen receptor is indirect evidence connecting estrogen receptor function, transcriptional repression, and E-cadherin expression in breast cancer. The dynamic nature of epigenetic regulation including changes in DNA methylation patterns, in expression and/or function of *trans*/acting factors and chromatin mediated effects, could explain the lack of uniformity in the *CDH1 *hypermethylation and the loss of E-cadherin expression observed at the immunohistochemical level.

## Competing interests

The author(s) declare that they have no competing interests.

## Authors' contributions

JRFC participated in the design of the study, in defining the casuistic used and helped to draft the manuscript. ECP carried out the molecular genetic studies, and drafted the manuscript. FAMN and FCQ carried out the immunohistochemistry analysis. CAR and SRR conceived of the study, participated in its design, performed the statistical analysis, and helped to draft the manuscript. All authors read and approved the final manuscript.

## Pre-publication history

The pre-publication history for this paper can be accessed here:



## Figures and Tables

**Table 1:** *CDH1 *promoter methylation distribution according to clinical variables and immunohistochemical analysis in 71 IDCs.

Variable	*CDH1 *methylation		p value*
	
	Presence N (%)	Absence N (%)	
Age			0,7842
≤50	19 (76%)	6 (24%)	
>50	33 (72%)	13 (28%)	
Lymph node^a^			0,5049
≤4	42 (75%)	14 (25%)	
>4	09 (64%)	5 (36%)	
Clinical Stage			0,5268
I	9 (90%)	1 (10%)	
IIA	16 (67%)	8 (33%)	
IIB	17 (68%)	8 (32%)	
IIIA	4 (100%)	0	
IIIB	6(75%)	2 (25%)	
Histologic grade			0,4282
I	2 (50%)	2 (50%)	
II	37 (76%)	12 (24%)	
III	13 (72%)	5 (28%)	
**Immunohistochemical**			
E-cadherin			0,1496
negative/reduced expression (0,1+,2+)	46 (77%)	14 (33%)	
positive (3+)	6 (55%)	5 (45%)	
Her-2			0,4339
negative (0,1+,2+)	47 (75%)	16 (25%)	
positive (3+)	5 (63%)	3 (37%)	
p53			1,0000
negative (0,1+)	42 (72%)	16 (28%)	
positive (2+,3+)	10 (77%)	3 (23%)	
ER			0,0301
negative/reduced expression (0,1+,2+)	37 (82%)	8 (18%)	
positive (3+)	15 (58%)	11(42%)	
PgR			0,3910
negative/reduced expression (0,1+,2+)	37 (77%)	11 (23%)	
positive (3+)	15 (65%)	8 (35%)	
Ki-67			0,0565
≤25%	27 (64%)	15 (36%)	
>25%	25 (86%)	4 (14%)	

* – p-value obtained from Fisher's exact test. Bonferroni correction was applied to statistical adjustment of each p-value (statistically significant p < 0,005); ^a ^70 patients contributed data.
